# Effectiveness of letters to patients with or without Cochrane blogshots on 10-year cardiovascular risk change among women in menopausal transition: 6-month three-arm randomized controlled trial

**DOI:** 10.1186/s12916-022-02555-2

**Published:** 2022-10-20

**Authors:** Slavica Jurić Petričević, Ivan Buljan, Dora Bjelanović, Nataša Mrduljaš-Đujić, Tanja Pekez, Mario Ćurković, Željko Vojvodić, Ivančica Pavličević, Matko Marušić, Ana Marušić

**Affiliations:** 1 Department of Pulmonary Diseases, University of Split Hospital Center, Spinčićeva 1, Split, Croatia; 2 Department of Research in Biomedicine and Health and Center for Evidence-based Medicine, University of Split School of Medicine, Šoltanska 2, Split, Croatia; 3 Department of Abdominal Surgery, University of Split Hospital Center, Spinčićeva 1, Split, Croatia; 4 Department of Family Medicine, University of Split School of Medicine, Šoltanska 2, Split, Croatia; 5 Family Practice Office, Kutina, Croatia; 6 Family Practice Office, Health Center of the Osijek-Baranja County, Osijek, Croatia; 7 Department of Family Medicine, JJ Strossmayer University School of Medicine, Osijek, Croatia; 8 Family Practice Office, Bijelo Brdo, Croatia; 9 University of Split, Ulica Ruđera Boškovića 31, 21000 Split, Croatia

**Keywords:** Cochrane blogshots, Educational intervention, CVD risk, Women

## Abstract

**Background:**

Health information and patient education on lifestyle changes may have a positive effect on the prevention of many chronic conditions, especially cardiovascular diseases (CVDs). We performed a parallel, three-arm randomized controlled trial (RCT) of 6-month educational intervention in a form of letters containing a reminder of the participant’s CVD risk with or without Cochrane blogshots to reduce CVD risk among women aged 45–65 with one or more known CVD risk factors.

**Methods:**

The control group received a letter about their CVD risk at the beginning of the trial. The intervention groups received the initial letter about their CVD risk and remainder letters about their CVD risk every 2 months, with or without Cochrane blogshots: (1) effect of calcium in the prevention of high blood pressure, (2) effect of reducing saturated fat acids in eating habits, and (3) effects of green and black tea in CVD prevention. The primary outcome was CVD risk reduction calculated as the difference between the baseline and 6-month score for a 10-year risk of fatal CVD according to the ACC/AHA guidelines.

**Results:**

After both interventions, CVD risk reduction was significantly higher compared to the control group (*P* < 0.001, Kruskal-Wallis *H* test). The number of participants who decreased their CV risk was 29% (20/70) in the control group, 69% (48/70) in the group receiving the reminder letters, and 70% (49/70) in the group receiving the reminder letters and blogshots. The number needed to treat to achieve risk reduction was 2.41 (95% CI = 1.77 to 3.78) for letters with a CVD risk reminder and 2.50 (1.81 to 4.03) for letters with a reminder and a blogshot. The group receiving reminder letters with Cochrane blogshots had a significant change in the category of CVD risk, mainly from high to moderate and from moderate to low CVD risk category.

**Conclusions:**

A simple and inexpensive intervention method in a form of letters reminding women about their CVD risk with or without providing additional health information in the form of Cochrane blogshots about interventions for important CVD risk factors may be effective in CVD management and could be considered by primary care providers.

**Trial registration:**

ClinicalTrials.gov, NCT04601558. Retrospectively registered on October 19, 2020

**Supplementary Information:**

The online version contains supplementary material available at 10.1186/s12916-022-02555-2.

## Background

Translation of health information to the patients is an important aspect of educational interventions in health care. One of the highly respected and high-quality sources of information for both the doctors and the patients is summaries of evidence synthesis—scientific abstracts and plain language abstracts, respectively, such as those produced by Cochrane. We have previously shown that plain language summaries of Cochrane systematic reviews increase knowledge of patients about health issues that is comparable to infographics of the same abstract [1]. Shortened versions of plain language summaries of Cochrane systematic reviews in the form of single-slide brief and focused information (blogshots) seem to be even more effective than plain language summaries in terms of change in comprehension of health information among patients/consumers [1].

An area where the translation of best evidence to the patients/consumers is important is chronic diseases, such as cardiovascular diseases (CVDs). They are the leading cause of morbidity and mortality worldwide, so the prevention of CVDs remains high on the agenda of any health care system [2, 3]. The focus is on the primary prevention of CVD: identifying and treating risk factors, including hypertension, dyslipidemia, diabetes, smoking, obesity, and physical inactivity [4]. Due to the misperception that females are “protected” against CVD, the risk of CVD in women is often underestimated [5]. The lack of awareness by women about their CVD risks represents a challenge to effective and timely patient management [6]. Several studies have shown that women receive suboptimal CVD preventive care, and gender disparities in recommendations for preventive therapy have been largely explained by the lower perceived danger of CVD risk in women despite the similar calculated risk for women versus men [7].

Lifestyle change programs have a beneficial effect on recurrent cardiovascular events because CVD is strongly associated with lifestyle, especially tobacco use, unhealthy dietary habits, physical inactivity, and psychosocial stress [8]. Recent studies have shown that patient education can contribute to changing behavior and improving compliance with the prescribed preventive and therapeutic measures [9, 10]. These educational interventions include clinical decision support, education, patient-involvement strategies, telephone and email support follow-up [11, 12], mobile health technology [13], and nurse-led educational intervention [14], significantly improving knowledge, drug adherence, and quality of life in patients with CVD.

To test the effectiveness of providing a summary of the best evidence for interventions for CVD risk in reducing CVD risk among women in menopausal transition who have one or more known cardiovascular risk factors, we performed a parallel, three-arm randomized controlled trial (RCT) in a 6-month educational intervention in a form of letters containing a reminder of the participant’s 10-year CVD risk with or without Cochrane blogshots about interventions directed to some CVD risk factors.

## Methods

### Trial design

This parallel, three-arm randomized controlled trial (RCT) tested the effect of a 6-month educational intervention in a form of letters in decreasing the 10-year CVD risk in women aged 45 to 65 with one or more known CVD risk factors. The letters reminded the participants of their own 10-year CVD risk, with or without included Cochrane blogshots.

### Participants

The inclusion criteria were (1) female sex; (2) age 45 to 65 years, which is the age of menopause transition, including postmenopause [15]; and (3) one or more CVD risk factors: overweight or obesity (body mass index (BMI) ≥ 25 kg/m^2^, and/or central obesity, i.e., waist circumference ≥ 88 cm), high blood pressure (systolic blood pressure ≥ 140 mmHg and/or diastolic blood pressure ≥ 90 mmHg), high blood cholesterol (≥ 5.2 mmol/L), and active smoking. The participants on antihypertensive therapy were eligible for the study.

Current CVD (ischemic heart disease, peripheral artery disease, and stroke), malignant diseases, serious systemic diseases, and/or mental diseases were the exclusion criteria.

The trial took place in family medicine offices in Croatia from February 1, 2018, to September 1, 2020. Family medicine offices are primary health care offices, i.e., the first step for patients to seek help for their health problems in the Croatian health care system, which ensures full national health coverage. Physicians working in family medicine offices are specialists in family medicine. One office was in the city of Split, the capital of the Split-Dalmatia County; one in the city of Osijek, the capital of the Osijek-Baranja County; and one in the city of Rijeka, the capital of the Primorje-Gorski Kotar County. An office from each of the following towns was the recruitment sites: Supetar (the island of Brač, Split-Dalmatia County), Bijelo Brdo (Vukovar-Srijem County), Kotoriba (Međimurje County), and Kutina (Sisak-Moslavina County). In each family medicine office, 30 participants were recruited. All participants involved in this trial had access to their family medicine office according to their place of residence. The first participant was recruited in February 2018, and the last participant was recruited in February 2020. The recruitment was performed by family medicine doctors in their medical offices. They also performed clinical measurements at the baseline and 6 months after the intervention, after sending the notification to the participants about their check-up appointment. The data from the two time points were sent to the primary investigator.

The Ethics Committee of the University of Split, School of Medicine, approved the study, No. 2181-198-03-94/10-11-0038 and No. 2181-198-03-04/19-0044. Written informed consent was obtained from all participants after they received the information about the study. The data were processed as pseudonymized (coded) data, following the General Data Protection Regulation (GDPR).

### Intervention

The participants were randomized into three parallel groups:

1. Control group: A month after recruitment, the participants received a letter that included the list of their own CVD risk factors and their 10-year risk of CVD, based on the data provided by the participants during recruitment. This was the only letter this group received.
2. Passive intervention group: The participants first received a letter with the same information as the control group. After this letter, the participants received a letter every 2 months, which reminded them of their own CVD risk factors and their 10-year risk of CVD. In total, this group received four letters at their home address during the trial.
3. Active intervention group: The participant first received the same letter as the other two groups. Every 2 months after that letter, they received a reminder about their own CVD risk factors and their 10-year risk of CVD, together with a Cochrane systematic review summary in the form of a blogshot. The topics in the blogshots were the following: (1) the effect of calcium in the prevention of high blood pressure [16], (2) the effect of reducing saturated fat acids in eating habits [17], and (3) the effects of green and black tea in the prevention of CVD [18]. In total, this group received four letters at their home address during the trial.

The examples of all types of letters sent to the participants in each group (in Croatian) are available in Additional file 1: Letters 1–5, including the Cochrane blogshots (in Croatian) used in the letters.

### Outcomes

At recruitment and at 6 months after the intervention, we measured the following clinical parameters: height, weight, BMI, waist and hips circumference, systolic and diastolic blood pressure, total serum cholesterol, high-density lipoprotein (HDL), low-density lipoprotein (LDL) cholesterol, triglycerides, and glucose.

The primary outcome measure was the change in the score between the 10-year risk of CVD at the beginning of the study and the 10-year risk of CVD after 6 months. The 10-year risk of fatal CVD was estimated using an online tool (https://www.cvriskcalculator.com/) based on the American College of Cardiology/American Heart Association (ACC/AHA) guidance [19]. The calculation is based on the following data collected from the study participants: age, gender, race, total and HDL cholesterol, systolic blood pressure, data about antihypertensive therapy, diabetes mellitus, and smoking status. The 10-year risk of fatal CVD was expressed as a percentage and was calculated at the beginning of the trial and at 6 months, after the participants received the last letter.

The secondary outcome measures were the changes in weight (in kg), body mass index (BMI), waist circumference (in cm), hip circumference (in cm), and smoking status (related to continued or excessive smoking) at 6 months post-intervention. The number of participants who changed their CVD risk category—low, moderate, and high [19]—was also calculated.

After recruitment, the participants first completed the pre-study questionnaire (in Croatian), which included (a) demographic data (available in Additional file 2), (b) Decisional Conflict Scale (DCS) [20–22], and (c) future time perspective (FTP) [23].

The DCS consists of 16 items rated on a 5-point Likert scale and measures an individual’s uncertainty toward a course of action. The score is calculated as a sum of items, divided by the number of items and multiplied by 25, allowing for a score range from 0 (no decisional conflict) to 100 points (extreme decisional conflict) [20, 21]. The Croatian version of the scale was previously validated [22].

The FTP is a 13-item scale from the Time Perspective Inventory [23]. It assesses how respondents focus on planning and achievement of future goals. The respondent answers using a 5-point Likert scale from 1 (does not refer to me) to 5 (refers to me completely), maximum score of 65. Higher scores indicate a greater future time perspective.

### Sample size

As there were no previous studies on this topic, we hypothesized that in the intervention group at the end of the trial, the mean CVD risk would be 6.0 and 8.0 in the control group, with a standard deviation of 3. With a study strength of 0.8 and alpha level of 0.01 (to take multiple comparisons into account), we used an online sample size calculator (https://epitools.ausvet.com.au/twomeansone), to estimate that we would need at least 53 participants per group (159 in total). To compensate for potential dropouts, we aimed to recruit 70 participants per trial arm.

### Randomization and blinding

Trial groups were formed by a random assignment, so that each respondent had one probability of falling into one of the three research groups (1:1:1) and each group of participants was exposed to only one of the study interventions. The generation of the random sequence was performed by a statistician who was not involved in the conduct of the trial, using https://www.randomizer.org/. Only the main investigator was aware of the allocation of participants into the study groups, and the participants, the family medicine doctors, or the statistician were not aware of the allocation of participants to trial arms. The family medicine doctors made physical exams and took all measurements of each eligible participant. The main investigator prepared all letters, sealed them in opaque envelopes, and sent them to the addresses collected from the medical records of the participants.

### Statistical analysis

Categorical data are presented as frequencies and percentages. Numerical variables did not follow a normal distribution and were presented as medians with IQR or with 95% confidence intervals (CIs). Due to the asymmetrical distribution of the variables, a parametric 2 × 2 factorial analysis could not be performed. As there is no non-parametric analog, the post-intervention differences between the three groups were compared by using the Kruskal-Wallis test with post hoc Conover-Iman test. To address multiple comparisons, we applied the Bonferroni correction to avoid type I error. The differences in CVD risk were made by subtracting the scores at the end of the trial with the ones from the baseline assessment, which sometimes resulted in negative scores. The results were expressed as a median difference in the score (with 95% CI). The changes in the number of participants who changed the category of their 10-year CVD risk after the interventions were tested using the McNemar *χ*^2^ test. The risk ratios and numbers needed to treat (NNTs) were calculated based on the number of participants who had a decrease in CVD risk after the interventions. We used linear regression to determine the variables that predicted the pre- and post-assessment changes. The results were expressed as unstandardized coefficients and *R*^2^. All analyses were performed by the R programming software (R Core Team, 2021).

### Trial registration

The trial was retrospectively registered on ClinicalTrials.gov on October 19, 2020 (trial registration number NCT04601558). The trial was retrospectively registered because of the lack of timely official access to the registry by the first author from the University Hospital of Split.

## Results

**Fig. 1:**
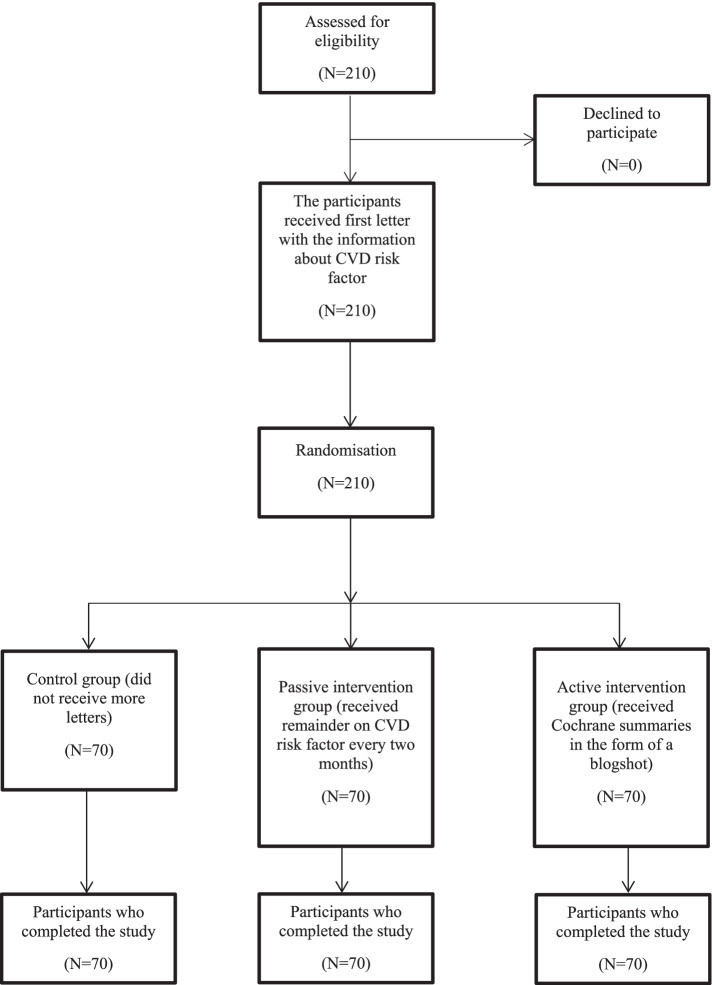
Flow of the participants in the trial

In total, 210 participants took part in the study (70 per group) with a median age of 58 years (IQR 52–62) (Fig. 1). There were no dropouts or missing data points in all groups. The majority of the participants had at least a high school education and were employed, married, and in postmenopausal reproductive status. Almost 90% of the participants gave birth at least once (Table 1). The majority of the participants had been prescribed antihypertensive therapy, and obesity, arterial hypertension, and high cholesterol were the most prevalent types of CVD risk (Table 1). Patients from all groups had a rather low decisional conflict and high future time perspective, regardless of the group (Table 1).

**Table 1:** Demographic characteristics (*n* (%)) of the trial participants

Variable	Overall (*n* = 210)	Control (*n* = 70)	Letters with CVD risk reminder (*n* = 70)	Letters with CVD risk reminder and Cochrane blogshot (*n* = 70)
Age (median, IQR)	58 (52 to 62)	58 (54 to 61)	59 (53 to 62)	58 (52 to 62)
Educational level				
Elementary school	45 (21.4)	17 (24.3)	15 (21.4)	13 (18.6)
High school	127 (60.5)	40 (57.1)	47 (67.1)	40 (57.1)
College	16 (7.6)	8 (11.4)	4 (5.7)	4 (5.7)
University	22 (10.5)	5 (7.1)	4 (5.7)	13 (18.6)
Work status				
Employed	101 (48.1)	26 (37.1)	34 (48.6)	41 (58.6)
Unemployed	52 (24.7)	23 (32.9)	18 (25.7)	11 (15.7)
Retired	57 (27.1)	21 (30.0)	18 (25.7)	18 (25.7)
Marital status				
Married	154 (73.3)	49 (70.0)	55 (78.6)	50 (71.4)
Not married	15 (7.1)	5 (7.1)	3 (4.3)	7 (10.0)
Divorced	17 (8.1)	6 (8.6)	4 (5.7)	7 (10.0)
Widowed	24 (11.4)	10 (14.3)	8 (11.4)	6 (8.6)
Reproductive status				
Premenopausal status	42 (20.0)	17 (24.3)	13 (18.6)	12 (17.1)
Perimenopausal status	14 (6.7)	6 (8.6)	5 (7.1)	3 (4.3)
Postmenopausal	154 (73.3)	47 (67.1)	52 (74.3)	55 (78.6)
Childbirths				
No	22 (10.5)	8 (11.4)	5 (7.1)	9 (12.9)
One	32 (15.2)	13 (18.6)	10 (14.3)	9 (12.9)
Two	112 (53.3)	37 (52.9)	38 (54.3)	37 (52.9)
Three or more	44 (21.0)	12 (17.1)	17 (24.3)	15 (21.4)
Antihypertensive therapy (yes)	145 (69.0)	43 (61.4)	55 (78.6)	47 (67.1)
Diabetes (yes)	30 (14.3)	8 (11.4)	14 (20.0)	8 (11.4)
Smoking (yes)	69 (32.9)	25 (35.7)	18 (25.7)	26 (37.1)
Type of cardiovascular risk				
Overweight	158 (75.2)	50 (71.4)	56 (80.0)	52 (74.3)
Diabetes	29 (13.8)	8 (11.4)	12 (17.1)	9 (12.9)
Arterial hypertension	149 (71.0)	43 (61.4)	58 (82.9)	48 (68.6)
High cholesterol	167 (79.5)	53 (75.7)	54 (77.1)	60 (85.7)
Sedentary lifestyle	86 (40.9)	24 (34.3)	28 (40.0)	34 (48.6)
Unhealthy eating habits	105 (50.0)	37 (52.9)	35 (50.0)	33 (47.1)
Smoking	66 (31.4)	25 (35.7)	16 (22.9)	25 (35.7)
10-year CVD risk score^a^ (%, median, IQR)	5.2 (3.1 to 8.9)	5.1 (2.8 to 7.3)	6.1 (3.7 to 10.3)	5.0 (3.0 to 8.9)
Decisional conflict score (median, IQR)^b^	25.8 (17.1 to 34.3)	25.0 (17.1 to 34.3)	28.1 (22.2 to 39.0)	25.0 (15.6 to 31.2)
*Informed* subscale	25.0 (25.0 to 25.0)	25.0 (25.0 to 33.0)	33.3 (25.0 to 33.3)	25.0 (25.0 to 25.0)
*Values clarity* subscale	25.0 (25.0 to 25.0)	25.0 (16.6 to 25.0)	25.0 (25.0 to 25.0)	25.0 (16.6 to 25.0)
*Support* subscale	25.0 (25.0 to 25.0)	25.0 (25.0 to 33.0)	25.0 (25.0 to 33.0)	25.0 (16.6 to 25.0)
*Uncertainty* subscale	25.0 (25.0 to 33.0)	25.0 (25.0 to 33.0)	33.3 (25.0 to 41.6)	25.0 (16.6 to 25.0)
*Effective decision* subscale	25.0 (25.0 to 25.0)	25.0 (25.0 to 25.0)	25.0 (25.0 to 31.2)	25.0 (18.7 to 25.0)
Future time perspective (median, IQR)^c^	50.5 (45.0 to 55.0)	51.0 (45.0 to 55.0)	49.0 (45.0 to 54.0)	51.0 (46.3 to 56.0)

*IQR* interquartile range, *CVD* cardiovascular risk, *ACC/AHH* American College of Cardiology/American Heart Association

^a^According to ACC/AHH guidelines [19], https://www.cvriskcalculator.com/

^b^Decisional Conflict Scale (DCS), score range 0 (no decisional conflict) to 100 points (extreme decisional conflict) [21]

^c^Future Time Perspective Scale, score range from 13 (low) to 65 (high future time perspective)

The median differences between the post- and pre-intervention CVD risk scores were greater for both intervention groups in comparison with the control group (Table 2). While the women in the control group had an increase in their post-intervention CVD risk (median difference = 0.55, 95% CI = 0.2 to 1.0), the group receiving reminder letters only or with Cochrane blogshots decreased their CVD risk (median difference = − 0.6%, 95% CI = − 1.0 to − 0.2 and median difference = 0.9%, 95% CI = − 1.5 to − 0.4, respectively). The results of the Kruskal-Wallis *H* test showed that there was a statistically significant difference between the three groups (*χ*^2^_2_ = 31.0, *P* < 0.001, with a mean rank score of 138.3 for the control group, 92.8 for the group receiving letters with reminders about their 10-year CVD risk, and 85.4 for the group receiving reminders and Cochrane blogshots). The Conover post hoc test comparison showed that the control group significantly differed from the other two groups, which did not differ between themselves.

**Table 2:** Pre-post intervention differences in cardiovascular (CVD) risk-related variables (median difference, 95% confidence interval) before and after interventions

	Overall (*n* = 210)	Control (*n* = 70)	Letters with CVD risk reminder (*n* = 70)	Letters with CVD risk reminder and Cochrane blogshots (*n* = 70)	*P*^a^
Weight (kg)	0 (0 to 0)	1 (0 to 2)	0 (− 2 to 3)	0 (− 3 to 0)	**0.002**^**b**^
Body mass index	0 (0 to 0)	0.2 (0.0 to 0.8)	0.0 (− 0.8 to 0.0)	0.0 (1.1 to 0.0)	0.004
Waist circumference (cm)	0 (− 1 to 0)	0 (0 to 1)	0 (0 to 0)	− 2 (− 2 to 0)	0.085
Hip circumference (cm)	0 (0 to 0)	0 (0 to 2)	− 1 (− 2 to 0)	− 1 (− 2 to 0)	**< 0.001**^**c**^
Systolic pressure (mmHg)	0 (0–0)	5 (0 to 5)	0 (− 10 to 0)	− 5 (− 13 to − 8)	**< 0.001**^**d**^
Diastolic pressure (mmHg)	0 (− 5 to 0)	0 (− 5 to 0)	0 (− 5 to 0)	0 (− 5 to 0)	0.286
Total cholesterol (mmol/L)	0 (0–0)	0.2 (0.0 to 0.3)	0.0 (− 0.4 to 0.0)	− 0.1 (− 0.3 to 0.1)	0.130
HDL cholesterol (mmol/L)	0.0 (0.0 to 0.1)	0 (0 to 0)	0.0 (0.0 to 0.1)	0.1 (0.0 to 0.2)	0.013
Non-HDL cholesterol (mmol/L)	0.0 (− 0.2 to 0.1)	0.2 (0.0 to 0.3)	− 0.3 (− 0.4 to 0.0)	− 0.3 (− 0.4 to 0.2)	0.008
LDL cholesterol (mmol/L)	0 (0 to 0)	0 (0 to 0)	0 (0 to 0)	0 (0 to 0)	NA
Triglycerides (mmol/L)	0 (0 to 0)	0 (0 to 0)	0 (0 to 0)	0 (0 to 0)	NA
Glucose (mmol/L)	0 (0 to 0)	0 (0 to 0)	0 (0 to 0)	0 (0 to 0)	NA
10-year CVD risk (%)	− 0.2 (− 0.4 to 0.0)	0.5 (0.2 to 1.0)	− 0.6 (− 1.0 to − 0.2)	− 0.9 (− 1.5 to − 0.4)	**< 0.001**^**e**^

^a^Kruskal-Wallis *H* test with Conover post hoc comparison. The Bonferroni correction was applied, and *P* value level was set at *α* = 0.05/15 = 0.003

^b^*χ*^2^_2_=19.4, *P* < 0.001; a mean rank score of 126.4 for the control group, 97.2 for the group receiving letters with the reminder about their 10-year CVD risk, and 92.9 for the group receiving reminder letters and Cochrane blogshots. Conover post hoc comparison: control group vs two intervention groups

^c^*χ*^2^_2_=16.0, *P* < 0.001; a mean rank score of 129.1 for the control group, 91.4 for the group receiving letters with reminders about their 10-year CVD risk, and 95.9 for the group receiving reminder letters and Cochrane blogshots. Conover post hoc comparison: control group vs two intervention groups

^d^*χ*^2^_2_=19.4, *P* < 0.001; a mean rank score of 131.6 for the control group, 92.5 for the group receiving letters with reminders about their 10-year CVD risk, and 92.4 for the group receiving reminder letters and Cochrane blogshots. Conover post hoc comparison: control group vs two intervention groups

^e^CVD risk was calculated according to the ACC/AHH guidelines [19], https://www.cvriskcalculator.com/. Kruskal-Wallis *H* test: *χ*^2^_2_ = 31.0, *P* < 0.001; a mean rank score of 138.3 for the control group, 92.8 for the group receiving letters with the reminder about their 10-year CVD risk, and 85.4 for the group receiving reminder letters and Cochrane blogshots. Conover post hoc comparison: control group vs two intervention groups

The change in the CVD risk was the result of significant changes in weight, hip circumference, and blood pressure, where the same differences were observed as for the change in CVD risk (Table 2, Additional file 3: Table S1).

The number of participants who decreased their CV risk was 29% (20/70) in the control group, 69% (48/70) in the group receiving the reminder letters, and 70% (49/70) in the group receiving the reminder letters and blogshots. The risk ratio for the CVD risk decrease after intervention between the active intervention (reminder letters and blogshots) and control groups was 0.41 (95% CI = 0.27 to 0.61), and NNT was 2.41 (95% CI = 1.77 to 3.78). The risk ratio between the passive intervention (reminder letters only) and control groups was 0.42 (0.28 to 0.63), and NNT was 2.50 (1.81 to 4.03).

We also assessed the changes in the category of the 10-year CVD risk. In the control group and the group that received the reminder letters, there were no significant changes in the categories at baseline and post-intervention assessment (Additional file 3: Table S1). In the group receiving the reminder letters with Cochrane blogshots, there were significant changes in their CVD risk category (McNemar *χ*^2^ test, *χ*^2^_3_ = 8.77, *P* = 0.032, Additional file 3: Table S1): out of 22 participants who had high risk at baseline, 7 changed to the moderate and 2 to the low CVD risk category. Out of 13 participants who had moderate CVD risk at baseline, 8 changed to the low and 1 to the high CVD risk category. In the group of participants with low baseline CVD risk (*n* = 35), 3 changed to the moderate and none to the high CVD risk category after the intervention.

In the linear regression analysis that included all pre-intervention measurements except CVD risk, the model explained 48.9% of the variance, but the only significant variable which predicted the pre-post difference in CVD risk score was the higher result on the *Effective decision* subscale of the Decisional Conflict Scale (Table 3). However, when entered as the only predictor in a new model, the predictor strength of the *Effective decision* subscale was 0.28 (standard error, SE = 0.12), explaining only 2.7% of the variance. Finally, when linear regression models with the *Effective decision* subscale as the only predictor of CVD risk difference were built for each trial group, it was significant only in the passive intervention group (*B* = 0.06, SE = 0.02, *R*^2^ = 0.08).

**Table 3:** Linear regression of predictors of greater post-intervention difference in cardiovascular disease (CVD) risk before and after the interventions

Variable	Level	Unstandardized	Standard error	*P*
(Intercept)		− 36.985	69.776	0.599
Age in years		− 0.044	0.144	0.762
Educational level	Ref: elementary school			
	High school	− 2.806	1.404	0.052
	College	1.312	1.082	0.232
	University	1.618	2.043	0.433
Work status	Ref: employed			
	Unemployed	− 1.367	0.781	0.088
	Retired	0.543	1.134	0.635
Marital status	Ref: married			
	Not married	− 0.604	1.069	0.575
	Divorced	1.581	2.413	0.516
	Widowed	1.279	1.614	0.433
Reproductive status	Ref: premenopausal status			
	Perimenopausal status	0.45	1.041	0.668
	Postmenopausal	1.009	1.261	0.428
Childbirths	Ref: no			
	One	− 0.636	2.243	0.778
	Two	0.404	1.125	0.721
	Three or more	1.229	1.179	0.304
Height (cm)		0.219	0.423	0.607
Weight (kg)		− 0.366	0.442	0.413
BMI		0.923	1.160	0.431
Waist circumference (cm)		0.083	0.085	0.334
Hip circumference (cm)		− 0.045	0.084	0.599
HDL cholesterol (mmol/L)		1.746	1.675	0.303
LDL cholesterol l (mmol/L)		− 0.396	0.627	0.531
Glucoses (mmol/L)		− 0.544	0.535	0.315
Number of cigarettes per day		0.122	0.084	0.152
Decisional conflict: *Informed* subscale		0.031	0.049	0.528
Decisional conflict: *Values clarity* subscale		− 0.092	0.053	0.091
Decisional conflict: *Support* subscale		0.046	0.029	0.120
Decisional conflict: *Uncertainty* subscale		− 0.049	0.047	0.302
Decisional conflict: *Effective decision* subscale		0.103	0.039	0.012

## Discussion

This randomized controlled trial showed that a simple intervention in the form of letters reminding patients about their 10-year CVD risk was an effective measure in family practice management of increased CVD risk. After receiving three written reminders from their family medicine doctor’s office every 2 months, women with one or more known CVD risk factors significantly decreased those risks. Both the letters with a simple reminder about their CVD risk and letters with Cochrane blogshots in addition to the reminder were successful interventions. Letters including Cochrane blogshots had greater odds for a greater reduction in the CVD risk.

### Strengths and limitations

The strengths of our study include a simple, inexpensive, and non-invasive assessment of CVD risk factors, a high rate of participant adherence, and the parallel, three-arm randomized controlled study design which reduces the chance of confounding. Furthermore, the study provides a strong piece of evidence of the usefulness of providing CVD risk information and ways to decrease it as a simple and cost-effective method in shared-decision making. The strength of the study is also the use of the ACC/AHA guidelines to calculate CVD risk, based on a full set of data collected from the study participants (age, gender, race, total and high-density lipoprotein (HDL) cholesterol, systolic blood pressure, data about antihypertensive therapy, diabetes mellitus, and smoking status) [19]. Previous studies have indicated that more individuals are recommended for the treatment according to the ACC/AHA guidelines than to other guidelines [24].

The main limitation of the study is its short-term nature. We cannot make conclusions whether the intervention would have long-term effects. We also tested only Cochrane blogshots as the simplest way of presenting the evidence synthesis about health interventions. Our study was not specifically designed to differentiate the effects of Cochrane blogshots over CVD risk reminders, and further studies are needed to investigate the separate effects of blogshots over risk reminders. We included women of specific age, from 45 to 65 years, in order to cover the full menopause transition from premenopause, menopause, and early postmenopause, which is the period for increased CVD risk for women [15]. This means that other reproductive outcomes associated with an increased CVD risk could not be evaluated, such as pre-eclampsia, hypertensive disorders of pregnancy, and gestational diabetes mellitus [15]. Finally, the study was performed in a single country in a health system that is publicly funded, provides universal health care coverage, and has family medicine as the basic point of primary health care. We balanced the geographical, cultural, and economic differences among family medicine settings by including family medicine offices from 6 different country regions, including southern coastal and island trials sites and northern sites, as well as sites in major cities and smaller towns.

### Interpretation

The results from our trial suggest that a simple and inexpensive intervention method in a form of letters reminding patients about their CVD risk, especially when accompanied by health information in the form of Cochrane blogshots about interventions for important CVD risk factors, can play an important role in CVD management. Cardiovascular risk decreased significantly in the intervention groups compared to the control group. The intervention in the form of Cochrane blogshots—short textual information about a systematic review on a simple graphic template—has been shown to have a positive effect on the comprehension of health information among different users [1]. It is possible that other forms of presenting health information about interventions for CVD risk factor would be more effective than blogshots, especially those containing more detailed information about the results of Cochrane systematic reviews. There are several arguments against this hypothesis. First, we showed that the readability of plain language summaries of Cochrane systematic reviews is low, almost twice that of the recommended reading level for health information [25]. Secondly, in randomized trials, Cochrane infographic summaries did not produce a better understanding compared to lay summaries among different users—health consumers, doctors, and medical students, although users had greater preference for visual information [26]. Finally, we also showed in a randomized trial that blogshots were better than plain language summaries in increasing understanding of health information and had higher preference among all users—health consumers, doctors, and medical students [1]. The findings from this study suggest that a simple information such as a reminder of CVD risk sent at regular intervals is a useful intervention for decreasing CVD risk among women in menopausal transition in the setting of family medicine offices, at least early after diagnosing the risk and starting its management. Also, Cochrane blogshots may be a useful tool to structure health information so that it can be easily accessed and understood by patients.

Providing reminders about CVD risk and information about high-quality evidence about post-health risks in this study can be considered as a part of shared decision-making (SDM) process [27, 28], in which decision aids can help patients with some chronic diseases in lifestyle changes. In the linear regression model for a greater postintervention change in the 10-year CVD risk, the only significant variable was the higher score on the decisional conflict subscale *Effective decision*, which explained only 2.7% of variance and was a significant predictor only in the group that received only the reminder letters. It is difficult to interpret these results, which would indicate that higher scores on the *Effective decision* subscale, which means lower belief in one’s own ability to complete tasks or one’s own belief in the ability to make a good decision regarding the course of management, were more likely to reduce their CVD risk. Those participants may have come to the trial with lower effective decision skills, and the letters with reminders helped them increase those skills. Further studies are needed to explore the significance of this finding. A recent Cochrane systematic review showed that people exposed to decision aids are better informed and have a more active role in decision making, as well as accurate risk perception [29, 30]. The interventions such as sharing electronic health records (EHRs) may improve quality of care by providing patients with their personal health information, also involving them as key stakeholders in the self-management of their health and disease [31, 32]. In our study, doctors were the source of information for the patient. In other studies, health care providers have delegated preventive activities to practice nurses who work independently and have their own consultation. In the study by Laurant et al., experiences of chronic illness care in patients with established CVD or at high CVD risk did not change after the implementation of a tailored program aiming at nurses’ counseling skills [33]. On the other hand, a Cochrane systematic review by Huntink et al. suggested that appropriately trained nurses can provide care of the same quality as primary care doctors and achieve similar health outcomes for patients [34]. While patient health outcomes were similar for nurses and doctors, patient satisfaction was higher with nurse-led care because nurses tended to provide longer consultations, give more information to patients, and recall patients more frequently than doctors. In the public health care system in Croatia and other similar systems in central and Eastern Europe, where there are not enough nurses with relevant training [35], and where doctors still have the traditional central place in the healthcare system, family medicine doctors are still the best point of delivery of SDM information to the patient.

The prevention of CVD remains high on the agenda in health care systems, especially prevention based on shared-decision making, and the main goal of contemporary preventive medicine is therefore to encourage behavior change. However, while behavior change often seems easy in the short run, it can be difficult to sustain. Our study evaluated the short-term effects of an intervention to decrease CVD risks, but a recent systematic review [36] showed that the effects of lifestyle changes on the reduction in CVD risk factors reached their highest point at 12 months of follow-up and then gradually decreased over time. This may reflect the fact that the longer-term intervention may be more effective in reducing CVD risks but only if patients remain highly adherent to the interventions. As we showed in our study, the patients with higher risk and thus with better awareness about their status may benefit the most from being reminded about their CVD risk and potential interventions to alleviate it. Their health behavior can be influenced by a simple intervention, which is easily applicable in the primary care setting, as well as in low-resource economic settings.

## Conclusions

We showed that a simple method of reminding patients about their CVD risk and providing high-quality evidence in the form of Cochrane blogshots may be effective in the short-term reduction of the CVD risk. This can be a significant step in achieving sustainable lifestyle change in patients with high CVD risk and a contribution to the growing need for a more active participation of patients and a better understanding of their own health risks. Further research is required to assess the impact of CVD reminders to patients in larger population-based studies with longer follow-ups, cost-effectiveness and acceptability of different models of health service delivery, influence on the decision-making process, and the potential added value of providing high-quality evidence about health interventions.

## Supplementary Information

**Additional file 1.** Letter 1. The first letter sent to all trial participants (control group and intervention groups) (in Croatian). Letter 2. The letter containing the reminder about the CVD risk, sent to the passive intervention group (in Croatian). Letter 3. The letter containing the reminder about the CVD risk and the blogshot about the effect of calcium in the prevention of high blood pressure, sent to the active intervention group (in Croatian). Letter 4. The letter containing the reminder about the CVD risk and the blogshot about the effect of effects of reducing saturated fat acids on the risk of CVD, sent to the active intervention group (in Croatian). Letter 5. The letter containing the reminder about the CVD risk and the blogshot about the effect of green and black tea on the prevention of CVD, sent to the active intervention group (in Croatian).

**Additional file 2.** Demographic questionnaire for trial participants (in Croatian).

**Additional file 3: Table S1.** Comparison between CVD risks related variables (median, 95% confidence interval) between three groups before and after intervention.

## Data Availability

The data obtained and used in this study are available from the corresponding author upon reasonable request.

## Abbreviations

ACC/AHA: American College of Cardiology/American Heart Association
BMI: Body mass index
CI(s): Confidence interval(s)
CVD(s): Cardiovascular disease(s)
DCS: Decisional Conflict Scale
EHR(s): Electronic health record(s)
FTP: Future time perspective
GDPR: General Data Protection Regulation
HDL: High-density lipoprotein
LDL: Low-density lipoprotein
NNT: Number needed to treat
RCT: Randomized controlled trial
SDM: Shared decision-making
