# Supplementary material for: Effectiveness of letters to patients with or without Cochrane blogshots on 10-year cardiovascular risk change among women in menopausal transition: 6-month three-arm randomized controlled trial
Source: BMC Med. 2022 Oct 20;20:381. doi: 10.1186/s12916-022-02555-2 (PMC9583570; doi:10.1186/s12916-022-02555-2)

Sveučilište u Splitu
Medicinski fakultet

Universitas studiorum
Spalatensis
Facultas Medica

Šoltanska 2, 21 000 Split,
Hrvatska
Tel: +385 (0)21 557 900
Fax: 021 557 625
e-mail: office@mefst.hr
web: www.mefst.hr

Additional file 1:

Letter 1: The first letter sent to all trial participants (control group and intervention groups) (in Croatian).

Poštovana gđo. ___________,

Na početku Vam se želim zahvaliti na pristanku za sudjelovanje u istraživanju koje provodimo. Zahvaljujem Vam u svoje osobno ime kao i u ime svih članova tima koji ulažu mnogo vremena i truda u istraživanje. Vaš doprinos nam mnogo znači i vjerujemo da ćemo i dalje nastaviti u dobrom smjeru.

Ovim prvim pismom Vas želimo upoznati s rizikom za bolesti srca i krvnih žila, koji kod Vas iznosi _____ %. Slijedi tablični prikaz mjera kojima se procjenjuje rizik, u kojem su istaknute one vrijednosti koje su više od referentnih (normalnih), utječu na rizik za bolesti srca i krvnih žila i samim time ga povećavaju.

**Demografski podatci**		**Kolesterol**		**Krvni tlak**		**Rizični čimbenici**	
Godine	_____	Ukupni	mmol/l	Sistolički	mmHg	Šećerna bolest	da/ne
Spol	žensko	HDL	mmol/l	Dijastolički	mmHg	Pušenje	da/ne
Rasa	bijela			Na terapiji	da/ne		

Referentne vrijednosti:
ukupni kolesterol < 5 mmol/l; HDL-kolesterol > 1.2 mmol/l;
sistolički krvni tlak < 120 mmHg; dijastolički krvni tlak < 80 mmHg.

Još jednom Vam se zahvaljujem na sudjelovanju u istraživanju.
Nadam se da ćemo i dalje moći računati na Vas.

S poštovanjem,

Slavica Jurić Petričević, dr. med.

Sveučilište u Splitu
Medicinski fakultet

Universitas studiorum
Spalatensis
Facultas Medica

Šoltanska 2, 21 000 Split,
Hrvatska
Tel: +385 (0)21 557 900
Fax: 021 557 625
e-mail: office@mefst.hr
web: www.mefst.hr

Letter 2: The letter containing the reminder about the CVD risk, sent to the passive intervention group (in Croatian).

Poštovana gđo. __________,

Ovim pismom želim Vas podsjetiti na rizik za bolesti srca i krvnih žila, koji kod Vas iznosi _____%. U tabličnom prikazu mjera kojima se procjenjuje rizik, istaknute su one vrijednosti koje su više od referentnih (normalnih) te utječu na rizik za bolesti srca i krvnih žila i samim time ga povećavaju.

**Demografski podatci**		**Kolesterol**		**Krvni tlak**		**Rizični čimbenici**	
Godine	_____	Ukupni	mmol/l	Sistolički	mmHg	Šećerna bolest	da/ne
Spol	žensko	HDL	mmol/l	Dijastolički	mmHg	Pušenje	da/ne
Rasa	bijela			Na terapiji	da/ne		

Referentne vrijednosti:
ukupni kolesterol < 5 mmol/l; HDL-kolesterol > 1.2 mmol/l;
sistolički krvni tlak < 120 mmHg; dijastolički krvni tlak < 80 mmHg.

Zahvaljujem Vam na sudjelovanju u istraživanju.
S poštovanjem,

Slavica Jurić Petričević, dr. med.

Sveučilište u Splitu
Medicinski fakultet

Universitas studiorum
Spalatensis
Facultas Medica

Šoltanska 2, 21 000 Split,
Hrvatska
Tel: +385 (0)21 557 900
Fax: 021 557 625
e-mail: office@mefst.hr
web: www.mefst.hr

Letter 3: The letter containing the reminder about the CVD risk and the blogshot about the effect of calcium in the prevention of high blood pressure, sent to the active intervention group (in Croatian).

Poštovana gđo. __________,

Ovim pismom želim Vas podsjetiti na rizik za bolesti srca i krvnih žila, koji kod Vas iznosi _____%. U tabličnom prikazu mjera kojima se procjenjuje rizik, istaknute su one vrijednosti koje su više od referentnih (normalnih) te utječu na rizik za bolesti srca i krvnih žila i samim time ga povećavaju.

**Demografski podatci**		**Kolesterol**		**Krvni tlak**		**Rizični čimbenici**	
Godine	_____	Ukupni	mmol/l	Sistolički	mmHg	Šećerna bolest	da/ne
Spol	žensko	HDL	mmol/l	Dijastolički	mmHg	Pušenje	da/ne
Rasa	bijela			Na terapiji	da/ne		

Referentne vrijednosti:
ukupni kolesterol < 5 mmol/l; HDL-kolesterol > 1.2 mmol/l;
sistolički krvni tlak < 120 mmHg; dijastolički krvni tlak < 80 mmHg.

Zahvaljujem Vam na sudjelovanju u istraživanju.
S poštovanjem,

Slavica Jurić Petričević, dr. med.

Kako bismo Vam pomogli u smanjenju rizika za bolesti srca i krvnih žila, u narednim pismima predstavit ćemo Vam neke znanstveno provjerene informacije o zdravlju. U nastavku pisma prikazujemo Vam **znanstvene dokaze o tome kako dodatni unos kalcija djeluje na krvni tlak.** Znanstvene dokaze sažela je organizacija **Cochrane** – globalna neovisna mreža znanstvenika, profesionalnih zdravstvenih radnika, pacijenata, skrbnika i ljudi zainteresiranih

Sveučilište u Splitu
Medicinski fakultet

Universitas studiorum
Spalatensis
Facultas Medica

Šoltanska 2, 21 000 Split,
Hrvatska
Tel: +385 (0)21 557 900
Fax: 021 557 625
e-mail: office@mefst.hr
web: www.mefst.hr

za zdravlje, okupljenih oko jednoga zajedničkog cilja – izrade i diseminacije Cochraneovih sustavnih pregleda. Cochrane sustavni pregledni članci su znanstvena izvješća u kojima su sva postojeća objavljena primarna istraživanja vezana za ljudsko zdravlje prikupljena, kritički analizirana i sintetizirana, a zatim recenzirana. Sustavni pregledi randomiziranih kontroliranih pokusa ključni su u primjeni medicine utemeljene na dokazima.

Za vas smo pripremili vrlo kratki sažetak članka u obliku slikovnog bloga.

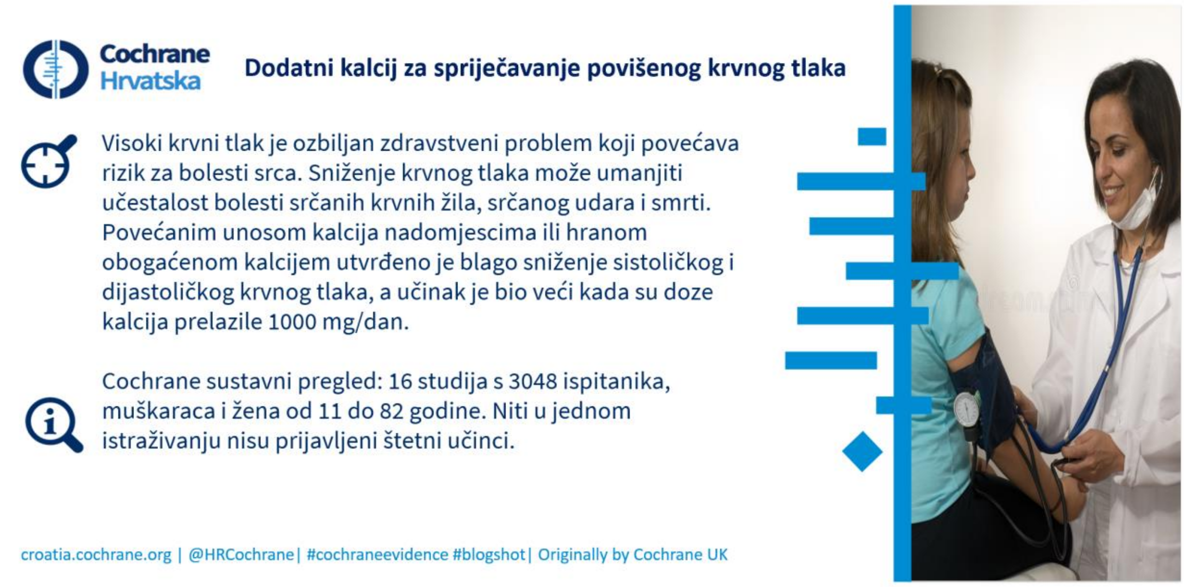

Sveučilište u Splitu
Medicinski fakultet

Universitas studiorum
Spalatensis
Facultas Medica

Šoltanska 2, 21 000 Split,
Hrvatska
Tel: +385 (0)21 557 900
Fax: 021 557 625
e-mail: office@mefst.hr
web: www.mefst.hr

Letter 4: The letter containing the reminder about the CVD risk and the blogshot about the effect of effects of reducing saturated fat acids on the risk of CVD, sent to the active intervention group (in Croatian).

Poštovana gđo. __________,

Ovim pismom želim Vas podsjetiti na rizik za bolesti srca i krvnih žila, koji kod Vas iznosi _____%. U tabličnom prikazu mjera kojima se procjenjuje rizik, istaknute su one vrijednosti koje su više od referentnih (normalnih) te utječu na rizik za bolesti srca i krvnih žila i samim time ga povećavaju.

**Demografski podatci**		**Kolesterol**		**Krvni tlak**		**Rizični čimbenici**	
Godine	_____	Ukupni	mmol/l	Sistolički	mmHg	Šećerna bolest	da/ne
Spol	žensko	HDL	mmol/l	Dijastolički	mmHg	Pušenje	da/ne
Rasa	bijela			Na terapiji	da/ne		

Referentne vrijednosti:
ukupni kolesterol < 5 mmol/l; HDL-kolesterol > 1.2 mmol/l;
sistolički krvni tlak < 120 mmHg; dijastolički krvni tlak < 80 mmHg.

Zahvaljujem Vam na sudjelovanju u istraživanju.
S poštovanjem,

Slavica Jurić Petričević, dr. med.

Kako bih Vam pomogla u smanjenju rizika za bolesti srca i krvnih žila, u narednim pismima predstavit ću Vam neke znanstveno provjerene informacije o zdravlju. U nastavku pisma prikazujem Vam **znanstvene dokaze o pozitivnom učinku smanjenja zasićenih masnih kiselina u prehrani.** Znanstvene dokaze sažela je organizacija **Cochrane** – globalna neovisna mreža znanstvenika, profesionalnih zdravstvenih radnika, pacijenata, skrbnika i ljudi zainteresiranih za zdravlje, okupljenih oko jednoga zajedničkog cilja – izrade i diseminacije

Šoltanska 2, 21 000 Split,
Hrvatska
Tel: +385 (0)21 557 900
Fax: 021 557 625
e-mail: office@mefst.hr
web: www.mefst.hr

Cochraneovih sustavnih pregleda. Cochrane sustavni pregledni članci su znanstvena izvješća u kojima su sva postojeća objavljena primarna istraživanja vezana za ljudsko zdravlje prikupljena, kritički analizirana i sintetizirana, a zatim recenzirana. Sustavni pregledi randomiziranih kontroliranih pokusa ključni su u primjeni medicine utemeljene na dokazima. Za Vas sam pripremila vrlo kratki sažetak članka u obliku slikovnog bloga.

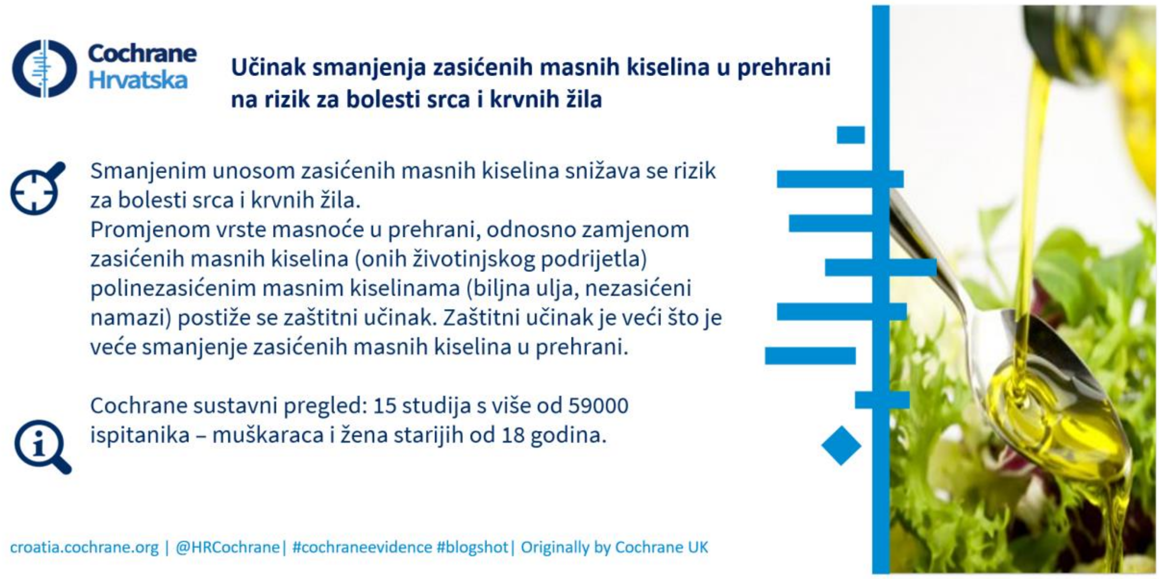

Sveučilište u Splitu
Medicinski fakultet

Universitas studiorum
Spalatensis
Facultas Medica

Šoltanska 2, 21 000 Split,
Hrvatska
Tel: +385 (0)21 557 900
Fax: 021 557 625
e-mail: office@mefst.hr
web: www.mefst.hr

Letter 5: The letter containing the reminder about the CVD risk and the blogshot about the effect of green and black tea on the prevention of CVD, sent to the active intervention group.

Poštovana gđo. __________,

Ovim pismom želim Vas podsjetiti na rizik za bolesti srca i krvnih žila, koji kod Vas iznosi _____%. U tabličnom prikazu mjera kojima se procjenjuje rizik, istaknute su one vrijednosti koje su više od referentnih (normalnih) te utječu na rizik za bolesti srca i krvnih žila i samim time ga povećavaju.

**Demografski podatci**		**Kolesterol**		**Krvni tlak**		**Rizični čimbenici**	
Godine	_____	Ukupni	mmol/l	Sistolički	mmHg	Šećerna bolest	da/ne
Spol	žensko	HDL	mmol/l	Dijastolički	mmHg	Pušenje	da/ne
Rasa	bijela			Na terapiji	da/ne		

Referentne vrijednosti:
ukupni kolesterol < 5 mmol/l; HDL-kolesterol > 1.2 mmol/l;
sistolički krvni tlak < 120 mmHg; dijastolički krvni tlak < 80 mmHg.

Zahvaljujem Vam na sudjelovanju u istraživanju.
S poštovanjem,

Slavica Jurić Petričević, dr. med.

Kako bih Vam pomogla u smanjenju rizika za bolesti srca i krvnih žila, u narednim pismima predstavit ću Vam neke znanstveno provjerene informacije o zdravlju. U nastavku pisma prikazujem Vam **znanstvene dokaze o pozitivnom učinku zelenog i crnog čaja u prevenciji srčano-žilnih bolesti.** Znanstvene dokaze sažela je organizacija **Cochrane** – globalna neovisna mreža znanstvenika, profesionalnih zdravstvenih radnika, pacijenata, skrbnika i ljudi zainteresiranih za zdravlje, okupljenih oko jednoga zajedničkog cilja – izrade i diseminacije Cochraneovih sustavnih pregleda. Cochrane sustavni pregledni članci su znanstvena izvješća u

Šoltanska 2, 21 000 Split,
Hrvatska
Tel: +385 (0)21 557 900
Fax: 021 557 625
e-mail: office@mefst.hr
web: www.mefst.hr

kojima su sva postojeća objavljena primarna istraživanja vezana za ljudsko zdravlje prikupljena, kritički analizirana i sintetizirana, a zatim recenzirana. Sustavni pregledi randomiziranih kontroliranih pokusa ključni su u primjeni medicine utemeljene na dokazima. Za Vas sam pripremila vrlo kratki sažetak članka u obliku slikovnog bloga.

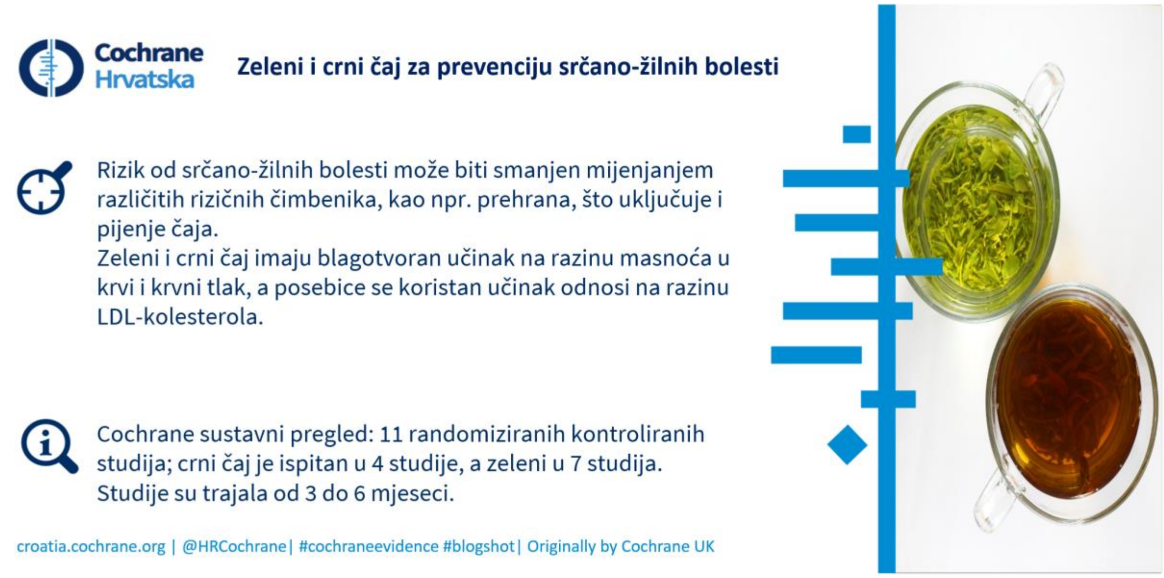

Supplement: Supplementary file 1 — Additional file 1. Letter 1. The first letter sent to all trial participants (control group and intervention groups) (in Croatian). Letter 2. The letter containing the reminder about the CVD risk, sent to the passive intervention group (in Croatian). Letter 3. The letter containing the reminder about the CVD risk and the blogshot about the effect of calcium in the prevention of high blood pressure, sent to the active intervention group (in Croatian). Letter 4. The letter containing the reminder about the CVD risk and the blogshot about the effect of effects of reducing saturated fat acids on the risk of CVD, sent to the active intervention group (in Croatian). Letter 5. The letter containing the reminder about the CVD risk and the blogshot about the effect of green and black tea on the prevention of CVD, sent to the active intervention group (in Croatian). [file 12916_2022_2555_MOESM1_ESM.pdf]
