# Supplementary material for: Effectiveness of letters to patients with or without Cochrane blogshots on 10-year cardiovascular risk change among women in menopausal transition: 6-month three-arm randomized controlled trial
Source: BMC Med. 2022 Oct 20;20:381. doi: 10.1186/s12916-022-02555-2 (PMC9583570; doi:10.1186/s12916-022-02555-2)
Supplement: Supplementary file 2 — Additional file 2. Demographic questionnaire for trial participants (in Croatian). [file 12916_2022_2555_MOESM2_ESM.docx]

**Additional file 2: Demographic questionnaire for trial participants (in Croatian).**

**Demografski podatci**

**Ime i prezime:** _________________________

**Adresa i broj telefona:** _________________________

**Datum rođenja:** _________________________

**Školska sprema:** _________________________

**Radni status:** _________________________

**Bračni status:** □udata/u stabilnoj vezi

□neudata

□razvedena

□udovica

**Reproduktivni status:** □zadnja menstruacija bila unutar 61 dana prije uključenja u istraživanje

□zadnja menstruacija bila između 61-365 dana prije uključenja u istraživanje

□zadnja menstruacija bila prije više od 365 dana prije uključenja u istraživanje

**Porodi (ako ih je bilo):** _________________________

**Ulazni podatci**

**Visina (cm):** __________ **Težina (kg):** __________ **BMI:** __________

**Opseg struka (cm):** __________ **Opseg bokova (cm):** __________

**Krvni tlak (mmHg):** __________ **Antihipertenzivna terapija (da/ne):** __________

**Ukupni kolesterol (mmol/l):** __________ **HDL kolesterol (mmol/l):** __________

**Non-HDL kolesterol (mmol/l):** __________ **LDL kolesterol (mmol/l):** __________

**Trigliceridi (mmol/l):** __________ **Glukoza (mmol/l):** __________

**Pušenje (da/ne; za pušače broj cigareta na dan):** __________

**Upitnik o znanju i ponašanjima vezanim za kardiovaskularne rizike**

**Koje kardiovaskularne rizike ima ispitanica?** *(ispunjava liječnik)*

□ dijabetes

□ hipertenziju

□ povišene vrijednosti kolesterola

□ prekomjernu tjelesnu težinu (BMI > 25)

□sjedilački način života*

□nezdrave prehrambene navike**

□pušenje

□nešto drugo _________________________

*Tjelesna aktivnost snažnijeg intenziteta manje od tri puta tjedno i manje od 60 minuta ukupno tjedno ili tjelesna aktivnost umjerenog intenziteta manje od tri puta tjedno i manje od 150 minuta ukupno tjedno.

**Nezdrave prehrambene navike: masnoće životinjskog podrijetla, nedostatak voća i povrća, previše soli u hrani.

**Je li netko iz Vaše obitelji već odlučio promijeniti životne i/ili prehrambene navike?**

□da □ne (ako da, tko _________________________)

**Jeste li prethodne odluke donosili bez pritiska od strane drugih osoba?**

□da □ne

**Ako je na Vas postojao pritisak, tko ga je provodio:**

□obitelj □prijatelji;

□liječnik obiteljske medicine □specijalist(i)

□netko drugi (ako da, tko _________________________)

**Znate li najvažnije koristi i rizike u odnosu na promjenu životnih i/ili prehrambenih navika?**

□da □ne

**Stupanj svog znanja/neznanja označite na ljestvici od 0 – 10, pri čemu 0 znači “nimalo”, a 10 “potpuno”.**

0 1 2 3 4 5 6 7 8 9 10

nimalo potpuno
