# Supplementary material for: Effectiveness of letters to patients with or without Cochrane blogshots on 10-year cardiovascular risk change among women in menopausal transition: 6-month three-arm randomized controlled trial
Source: BMC Med. 2022 Oct 20;20:381. doi: 10.1186/s12916-022-02555-2 (PMC9583570; doi:10.1186/s12916-022-02555-2)
Supplement: Supplementary file 3 — Additional file 3: Table S1. Comparison between CVD risks related variables (median, 95% confidence interval) between three groups before and after intervention. [file 12916_2022_2555_MOESM3_ESM.docx]

**Additional file 3:**

**Table S1. Comparison between CVD risks related variables (median, 95% confidence interval) between three groups before and after intervention**

| **Variable before** | **Overall (N=210)** | **Control (n=70)** | **Passive intervention (n=70)** | **Active intervention (n=70)** | **P*** |
| --- | --- | --- | --- | --- | --- |
| Height (cm) | 163 (162-164) | 163 (159 to 163) | 162 (160 to 164) | 164 (161 to 164) | 0.186 |
| Weight (kg) | 79 (77-80) | 78 (72-80) | 78 (74-82) | 80 (77-83) | 0.662 |
| BMI | 29.4 (28.4-30.8) | 29.4 (27.5 to 31.0) | 30.4 (28.3 to 31.2) | 29.2 (27.1 to 31.6) | 0.754 |
| Waist circumference | 97 (95-100) | 97 (91-100) | 100 (94-103) | 96 (89-97) | 0.657 |
| Hip circumference | 109 (107-111) | 108 (103-110) | 111 (104-111) | 108 (105-111) | 0.216 |
| Systolic pressure (mmHg) | 130 (130-135) | 130 (120-130) | 135 (130-140) | 130 (122-135) | 0.047 |
| Diastolic pressure (mmHg) | 80 (80-80) | 80 (80-80) | 80 (80-85) | 80 (80-85) | 0.677 |
| Total Cholesterol (mmol/l) | 5.95 (5.80-6.20) | 6.15 (5.50-6.45) | 5.75 (5.50-6.05) | 5.90 (5.50-6.10) | 0.534 |
| Total cholesterol (mg/dl) | 230 (224-240) | 238 (213-249) | 222 (213-234) | 228 (214-234) | 0.529 |
| HDL cholesterol (mmol/l) | 1.5 (1.3-1.6) | 1.4 (1.3-1.35) | 1.4 (1.4-1.5) | 1.6 (1.4-1.6) | 0.226 |
| HDL cholesterol (mg/l) | 58 (54-62) | 58 (50-62) | 54 (54-58) | 62 (54-62) | 0.226 |
| Non-HDL cholesterol (mmol/l) | 4.4 (4.2-4.6) | 4.6 (3.9-4.9) | 4.3 (3.9-4.5) | 4.4 (4.2-4.7) | 0.575 |
| LDL cholesterol (mmol/l) | 3.7 (3.4-3.9) | 3.6 (3.2-4.0) | 3.5 (3.2-3.9) | 3.7 (3.4-3.9) | 0.636 |
| Triglycerides (mmol/l) | 1.4 (1.3-1.6) | 1.5 (1.2-1.7) | 1.5 (1.2-1.6) | 1.4 (1.2-1.7) | 0.779 |
| Glucoses (mmol/l) | 5.3 (5.2-5.4) | 5.3 (5.0-5.4) | 5.3 (5.0-5.4) | 5.3 (5.0-5.5) | 0.515 |
| Number of cigarettes per day (for smokers) | 10 (10-10) | 10 (10-15) | 10 (10-10) | 10 (6-20) | 0.309 |
| CV Risk (ACC/AHH guidelines) | 5.15 (4.70-6.10) | 5.0 (3.9-6.9) | 6.1 (4.6-7.0) | 5.0 (3.9-6.9) | 0.302 |
| Future perspective | 51.(49 to 51) | 51 (47 to 52) | 49 (47 to 50) | 51 (47 to 51) | 0.113 |
| Personal incompetence | 18 (16 to 21) | 18.5 (14.5 to 22.9) | 19.5 (16.5 to 26.0) | 17.0 (14.5 to 20.0) | 0.412 |
| Decisional conflict total score | 25.8 (25.0 to 26.5) | 25.0 (18.7 to 25.0) | 28.1 (25.0 to 29.6) | 25.0 (17.1 to 25.0)‡ | 0.026 |
| Subscore informed | 25.0 (25.0 to 25.0) | 25.0 (25.0 to 33.0) | 33.3 (25.0 to 33.3) | 25.0 (25.0 to 25.0) | 0.055 |
| Subscore: Values clarity | 25.0 (25.0 to 25.0) | 25.0 (16.6 to 25.0) | 25.0 (25.0 to 25.0) | 25.0 (16.6 to 25.0) | 0.186 |
| Subscore: support | 25.0 (25.0 to 25.0) | 25.0 (25.0 to 33.0) | 25.0 (25.0 to 33.0) | 25.0 (16.6 to 25.0) | 0.719 |
| Subscore: uncertainty | 25.0 (25.0 to 33.0) | 25.0 (25.0 to 33.0) | 33.3 (25.0 to 41.6) | 25.0 (16.6 to 25.0)† | **0.007** |
| Subscore: effective decision | 25.0 (25.0 to 25.0) | 25.0 (25.0 to 25.0) | 25.0 (25.0 to 31.2) | 25.0 (18.7 to 25.0)‡ | 0.029 |
| **After** |  |  |  |  |  |
| Height (cm) | 163 (162-164) | 163 (159-163) | 162 (160-164) | 164 (161-164) | 0.186 |
| Weight (KG) | 80 (77-80) | 80 (74-82) | 80 (75-82) | 80 (75-84) | 0.949 |
| BMI | 29.7 (28.4-30.5) | 29.7 (26.7-31.8) | 30.1 (28.4-31.6) | 29.0 (27.5-30.9) | 0.824 |
| Waist circumference | 97 (95-100) | 98 (91-101) | 98 (95-103) | 95 (92-100) | 0.657 |
| Hip circumference | 109 (107-111) | 108 (105-112) | 112 (107-115) | 108 (105-110) | 0.375 |
| Systolic pressure (mmHg) | 130 (130-135) | 135 (130-140) | 130 (130-135) | 130 (125-135) | 0.113 |
| Diastolic pressure (mmHg) | 80 (80-80) | 80 (80-90) | 80 (80-75) | 80 (80-80) | 0.342 |
| Total Cholesterol (mmol/l) | 5.9 (5.6-6.1) | 6.1 (5.6-6.6) | 5.7 (5.4-6.1) | 6.0 (5.6-6.1) | 0.283 |
| Total cholesterol (mg/dl) | 228 (217-236) | 236 (217-255) | 220 (209-236) | 232 (217-236) | 0.277 |
| HDL cholesterol (mmol/l) | 1.6 (1.5-1.7) | 1.5 (1.4-1.6) | 1.6 (1.5-1.7) | 1.7 (1.6-1.9) | 0.021 |
| HDL cholesterol (mg/l) | 62 (58-66) | 66 (58-73) | 62 (58-66) | 66 (58-73) | 0.021 |
| Non-HDL cholesterol (mmol/l) | 4.3 (4.1-4.5) | 4.5 (4.1-5.0) | 4.0 (3.8-4.5) | 4.3 (4.0-4.6) | 0.094 |
| LDL cholesterol (mmol/l) | 3.7 (3.5-4.0) | 3.5 (3.2-4.1) | 3.6 (3.3-3.9) | 4.0 (3.6-4.3) | 0.113 |
| Triglycerides (mmol/l) | 1.3 (1.2-1.5) | 1.3 (1.2-1.7) | 1.4 (1.2-1.7) | 1.3 (1.1-1.7) | 0.854 |
| Glucoses (mmol/l) | 5.2 (5.1-5.3) | 5.2 (5.0-5.3) | 5.2 (5.0-5.5) | 5.1 (5.0-5.4) | 0.819 |
| CV Risk (ACC/AHH guidelines) | 4.6 (4.2-5.3) | 4.8 (4.0-7.2) | 5.2 (4.0-6.3) | 4.2 (3.1-5.3) | 0.123 |
